# Correlation of the clinical parameters with sonographic findings of hemorrhagic cystitis in pediatric hematooncology patients

**DOI:** 10.1186/s40064-015-1380-1

**Published:** 2015-10-06

**Authors:** In Kyung Youn, Soo Ah Im, Jae Wook Lee, Nak Gyun Chung, Bin Cho

**Affiliations:** Department of Radiology, Seoul St. Mary’s Hospital, College of Medicine, The Catholic University of Korea, 222 Banpo-daero, Seocho-gu, Seoul, 06591 Republic of Korea; Department of Pediatrics, Seoul St. Mary’s Hospital, College of Medicine, The Catholic University of Korea, 222 Banpo-daero, Seocho-gu, Seoul, 06591 Republic of Korea

**Keywords:** Cystitis, Sonography, Transplantation

## Abstract

To find a relationship between clinical and sonographic appearance of hemorrhagic cystitis (HC) in pediatric hematooncology patients. Clinical and sonographic findings of 31 children (M:F = 18:13; mean age, 12.7 years) with HC in pediatric hematooncology patients were reviewed. For each patient, the onset of HC after transplantation, use of bladder-toxic agent, presence of BK viruria, and duration of disease were reviewed. Sonographic findings including bladder wall thickness (BWT), the type of bladder wall thickening (nodular vs. diffuse), occurrence of hydronephrosis or pyelonephritis were reviewed. We analyzed sonographic appearance and clinical manifestations of HC. HC occurred within 4 months after HSCT/BMT. 27 patients (87.0 %) were positive for BK viruria and 24 patients (77.4 %) took bladder-toxic agents. On sonography, nodular type bladder wall thickening was more frequent (54.8 %), and BWT was thicker in this group (*p* = 0.003). There was a positive correlation between the BWT on initial sonography and duration of cystitis (*r*^2^ = 0.340). Hydronephrosis developed in 25.8 % of patients with HC, and as HC persisted longer, hydronephrosis occurred more (*p* = 0.004). In patients with HC after HSCT/BMT, the BWT on initial sonography correlates well with the duration of cystitis. And, longer time of HC develops the risk of hydronephrosis.

## Background

Hemorrhagic cystitis (HC) is bleeding of the urinary bladder that results when the uroepithelium is damaged by toxins, drugs, radiation, viruses, or bacteria. The reported incidence in pediatric hemato-oncology patients undergoing hematopoietic stem cell transplantation (HSCT)/bone marrow transplantation (BMT) ranges from 10 to 70 % depending on the definition and grade (Gorczynska et al. 2005; Hassan et al. 2007; Vogeli et al. 1999; Vose et al. 1993). Persistent or severe HC is often associated with significant morbidity and may prolong the hospitalization of these patients (Hale et al. 2003).

Risk factors include bladder-toxic agents in the conditioning regimen for BMT or HSCT, and viruses or bacteria since the patient is immunosuppressed (Decker et al. 2009). Oxazaphosphorine compounds such as cyclophosphamide and ifosfamide are well-known causes of HC. Cyclophosphamide and ifosfamide are metabolized into acrolein, which causes uroepithelial damage and hemorrhage. Hematuria usually occurs within 48 h of using these drugs (Manikandan et al. 2010; Stillwell and Benson 1988; Talar-Williams et al. 1996). Busulfan and alkylating agents like thiotepa, temozolomide, and 9-nitrocamptothecin (a topoisomerase I inhibitor) can also cause HC (Islam et al. 2002).

Pediatric hemato-oncology patients are also susceptible to developing infectious HC because they are immunocompromised. BK polyomavirus, adenovirus types 7, 11, 34, and 35, cytomegalovirus (CMV), JC virus, and herpes virus have all been reported to cause HC (Erard et al. 2004). BK virus is ubiquitous and prevalent in the pediatric population. After the initial infection, BK virus is thought to remain dormant and asymptomatic in the kidneys and other organs. When patients become immunocompromised, BK virus can be reactivated, which may lead to HC (Leung et al. 2005).

In hemato-oncology patients who have been given cyclophosphamide, the sonographic findings of HC include diffuse or focal thickening of the urinary bladder (McCarville et al. 2000; Suzuki et al. 1988). Schechter et al. reported the sonographic presentation of HC associated with BK virus following HSCT; it was characterized as multiple small mural nodules in a background of diffuse bladder wall thickening (Schechter et al. 2010).

To the best of our knowledge, however, the relationship between the sonographic appearance and clinical parameters of HC has not been reported.

Therefore, this study examined the relationships between the sonographic findings and clinical manifestations of severe HC because it is often associated with significant morbidity and may result in prolonged hospitalization in pediatric hemato-oncology patients. We examined correlations between the sonographic findings and clinical parameters, such as disease duration or hydronephrosis, in patients with BK viruria or patients who received any bladder-toxic agent. We also analyzed the bladder wall thickness (BWT) and type of bladder wall thickening and its association with prognosis, duration of disease, and complications of HC such as hydronephrosis or pyelonephritis.

## Results

Thirty-one children were included in the study (M:F ratio = 18:13; mean age = 12.7 years [range 5–17 years]) and HC occurred within 4 months after HSCT/BMT (mean = 34.5 days [range 7–111 days]). Table 1 summarizes the clinical parameters and sonographic findings of the 31 patients. The mean duration of cystitis was 46.6 days (range 4–236 days).

**Table 1:** Clinical informations and sonographic findings of the patients enrolled in this study

Patient number	Sex	Age	Known disease	BK viruria (y/n)	Bladder toxic agents	Use of Mesna (y/n)	Number of dates occurrence of HC after transplantation	Duration of HC (days)	BWT (mm)	Type of BWT (D/N)	Hydronephrosis	Use of cidofovir (y/n)
1	M	17	CML	n	B, C	y	32	52	10.8	D	–	n
2	F	11	ALL	n	C	y	27	58	21.0	N	y	n
3	M	12	ALL	y	–	–	38	43	4.1	D	–	n
4	M	14	AML	y	–	–	111	13	17.9	N	–	n
5	M	5	AML	y	–	–	86	7	11.1	N	–	y
6	M	16	AML	y	–	–	30	11	22.3	N	–	n
7	F	16	AA	y	–	–	48	66	10.1	N	–	n
8	M	16	DLBL	y	C	y	29	71	10.9	N	–	y
9	F	13	AML	y	B	–	43	24	13.7	N	–	y
10	M	16	ALL	y	C	y	31	21	6.2	D	–	n
11	M	8	ALL	y	C	y	36	65	12.0	D	y	n
12	M	12	ALL	y	C	y	25	39	11.0	D	–	y
13	M	16	AML	y	B	–	30	30	31.0	N	–	y
14	F	11	CML	y	B,C	y	21	52	15.7	N	–	y
15	M	15	ALL	y	C	y	31	25	10.3	D	–	y
16	F	7	AA	y	C	y	25	44	16.4	D	y	n
17	M	15	AA	y	C	y	29	53	12.7	N	y	n
18	M	5	ALL	y	C	y	25	10	10.9	D	–	n
19	M	16	ALL	y	C	y	7	236	43.0	N	–	y
20	F	13	ALL	y	C	y	26	34	11.8	D	y	n
21	M	13	AA	y	C	y	38	52	10.4	D	–	y
22	M	15	AML	y	B	–	33	16	8.2	D	–	n
23	M	17	CML	y	B, C	y	26	117	40.0	N	y	n
24	F	17	ALL	y	C	y	29	7	11.5	N	–	n
25	F	12	ALL	y	B	–	46	72	9.1	D	y	y
26	F	11	ALL	y	C	y	30	4	9.4	N	–	n
27	F	14	ALL	y	B	–	34	36	11.3	N	–	n
28	M	13	AML	y	B, C	y	35	93	12.2	N	–	y
29	F	7	AML	y	B	–	40	10	13.2	N	–	n
30	F	9	ALL	n	–	–	78	40	7.6	D	y	n
31	F	12	AA	n	–	–	28	43	7.4	N	–	n
Mean		12.7					34.5	46.6	14.3			

*CML* chronic myeloid leukemia, *ALL* acute lymphocytic leukemia, *AML* acute myeloid leukemia, *AA* aplastic anemia, *DLBL* diffuse large B cell lymphoma, *B* busulfan, *C* cyclophosphamide, N nodular type of bladder wall thickening, *D* diffuse type of bladder wall thickening, *y* yes, *n* no

There was a significant positive correlation between the BWT on initial sonography and the duration of disease (*r*^*2*^ = 0.340).

Classifying patients by the type of bladder wall thickening, the nodular type was more frequent and was present in 17 patients (54.8 %) with HC. The nodular type BWT (mean 17.8 mm) was significantly (*p* = 0.003) thicker than the diffuse type (mean = 10.0 mm). The mean disease duration, presence of hydronephrosis, and use of bladder-toxic agent or BK viruria did not differ significantly between the two groups (Table 2).

**Table 2:** Analysis of sonographic findings (mean BWT, hydronephrosis), clinical symptom (mean symptom duration of HC) and clinical conditions related to HC (use of bladder-toxic agent, BK viruria) between two groups classified as diffuse type HC group and nodular type HC group

	Diffuse type (*n* = 14)	Nodular type (*n* = 17)	*p*
Mean BWT (mm)	10.0	17.8	0.003
Mean duration of disease (days)	37.1	59.4	0.399
Hydronephrosis (n)	4 (28.6 %)	4 (23.5 %)	0.830
Bladder-toxic agent (n)	9	13	0.570
BK viruria (n)	12 (85.7 %)	15 (88.2 %)	0.622

*BWT* bladder wall thickness

We also classified patients by the presence of BK viruria. Twenty-seven patients (87.0 %) were positive for BK virus on urine PCR. The pattern of bladder wall thickening, mean BWT, presence of hydronephrosis, and mean duration of disease in HC did not differ significantly between BK virus-positive and -negative groups (Table 3).

**Table 3:** Analysis of sonographic findings (the type of bladder wall thickening, mean BWT) and clinical symptom (mean symptom duration of HC) between two groups classified as positive BK viruria group and negative BK viruria group

	BK viruria (+) (*n* = 27)	BK viruria (−) (*n* = 4)	*p*
Diffuse type (n)	12 (44.4 %)	2 (50.0 %)	0.622
Nodular type (n)	15 (55.6 %)	2 (50.0 %)	
Mean BWT (mm)	14.7	11.7	0.263
Mean duration of disease (days)	49.5	48.2	0.536

*BWT* bladder wall thickness

We also subdivided the patients according to the use of a bladder-toxic agent. Twenty-four patients (77.4 %) were given bladder-toxic agents such as cyclophosphamide, ifosfamide, and busulfan as a conditioning regimen before transplantation, while seven patients did not take bladder-toxic agents. The pattern of bladder wall thickening, mean BWT, BK viruria, presence of hydronephrosis, and mean duration of disease in HC did not differ significantly between the two groups (Table 4).

**Table 4:** Analysis of sonographic findings (the type of bladder wall thickening, mean BWT), clinical symptom (mean symptom duration of HC) and clinical condition related to HC (BK viruria) between two groups classified as bladder-toxic agent taken group and not taken group

	Bladder-toxic agent (+) (*n* = 24)	Bladder-toxic agent (−) (*n* = 7)	*p*
Diffuse type (n)	11 (45.8 %)	3 (42.9 %)	0.617
Nodular type (n)	13 (54.2 %)	4 (57.1 %)	
BK viruria (n)	22 (91.7 %)	5 (71.2 %)	0.212
Mean BWT (mm)	15.1	11.5	0.202
Mean duration of disease (days)	54.5	31.9	0.267

*BWT* bladder wall thickness

**Fig. 1:**
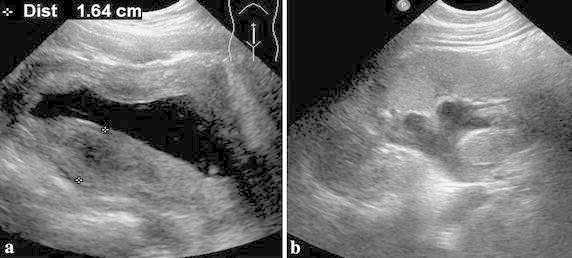
An 8-year-old girl with hemorrhagic cystitis (15th day after hematopoietic stem cell transplantation for aplastic anemia). **a** Sonography of the bladder shows diffuse type wall thickening of bladder. **b** Hydronephrosis developed at 23th day after the onset of hemorrhagic cystitis

Hydronephrosis developed in eight patients with HC (Table 5; Fig. 1). Equal numbers of patients with hydronephrosis had diffuse and nodular type wall thickening. The mean BWT on initial sonography was 16.6 mm in patients with hydronephrosis and 13.5 mm in those without hydronephrosis. The occurrence of hydronephrosis was not correlated with BWT (*p* = 0.362). The mean duration of disease was significantly (*p* = 0.004) longer in patients with hydronephrosis (86.3 days) than in patients without hydronephrosis (36.5 days).

**Table 5:** Analysis of sonographic findings (the type of bladder wall thickening, mean BWT) and clinical symptom (mean symptom duration of HC) between two groups classified as hydronephrosis group and no hydronephrosis group

	Hydronephrosis (+) (*n* = 8)	Hydronephrosis (−) (*n* = 23)	*p*
Diffuse type (n)	4	10	0.534
Nodular type (n)	4	13	
Mean BWT (mm)	16.6	13.5	0.362
Mean duration of disease (days)	86.3	36.5	0.004

*BWT* bladder wall thickness

One of the eight patients (12.5 %) who developed hydronephrosis underwent a percutaneous nephrostomy for severe obstructive nephropathy. Two of the eight patients (25.0 %) who developed hydronephrosis developed pyelonephritis.

## Discussion

Hemorrhagic cystitis can be classified as early or late onset by the onset time after transplantation. Early onset HC occurs within the first 2–3 days after transplantation and is thought to be a complication of thrombocytopenia and bladder-toxic agents in the conditioning regimen, such as cyclophosphamide, ifosfamide, or busulfan. Late-onset HC usually occurs several weeks after transplantation and is thought to be immune-related. An immunocompromised state (depressed cellular immunity, donor T-cell depletion, or graft-versus-host disease) may lead to viral infections, such as BK virus, JC virus, CMV and adenovirus 11, which may result in HC (Decker et al. 2009).

The mechanism of drug-induced early onset HC has been reported. Oxazaphosphorine compounds such as cyclophosphamide and ifosfamide are commonly used for myeloablative conditioning before transplantation. Cyclophosphamide is broken down into hydroxycyclophosphamide in hepatic microsomal cells, and then hydroxycyclophosphamide is converted into aldophosphamide by target cells. The aldophosphamide is converted into phosphoramide mustard, which is the active antineoplastic metabolite, and acrolein, which has no significant antineoplastic activity, but is toxic to the urothelium (Schoenike and Dana 1990). Similarly, ifosfamide, which is a synthetic analogue of cyclophosphamide, is metabolized to iphosphoramide mustard and acrolein (Manikandan et al. 2010). The urinary bladder can be damaged with prolonged exposure of the urothelium to acrolein (Ribeiro et al. 2002). Acrolein triggers the release of several inflammatory mediators, such as tumor necrosis factor-alpha, interleukin-1 beta, and endogenous nitric oxide (Ribeiro et al. 2002). These inflammatory mediators can result in bladder mucosal edema, vascular dilatation, and increased capillary fragility, which may cause urothelial hemorrhage. In chronic cases, progressive fibrosis of the wall can result in a small fibrotic non-compliant bladder (Kimura et al. 1998). Mesna (2-mercaptoethansulfonate-natrium; Uromitexan^®^) is an organosulfur compound that is used as an adjuvant in cancer chemotherapy with cyclophosphamide and ifosfamide. Mesna assists in detoxifying acrolein when the sulfhydryl group of the former reacts with the vinyl group of the latter. Hematuria usually occurs within 48 h of using these drugs (Stillwell and Benson 1988). Busulfan, an alkyl sulfonate compound used to treat chronic granulocytic leukemia, causes HC in about 24 % of patients (Ringden et al. 1994). However, the role of busulfan and its metabolites is not completely known. Other alkylating agents like thiotepa, temozolomide, and 9-nitrocamptothecin (a topoisomerase I inhibitor) have also been reported to cause HC (Islam et al. 2002).

The BK polyomavirus, adenovirus types 7, 11, 34, and 35, cytomegalovirus, JC virus, and herpes virus have been reported to cause HC (Erard et al. 2004).

Human BK polyomavirus can cause HC after HSCT. Human BK polyomavirus (or polyomavirus hominis 1) is ubiquitous and more than 80 % of the adult population has been exposed to it. After a primary infection that is usually asymptomatic, the virus remains dormant in the urinary tract (including the kidneys and uroepithelium of the bladder), lymphoid tissues, and circulating leucocytes. In patients with impaired immunity (such as pregnancy, diabetes, or the elderly) or with depressed cellular immunity, reactivation can occur with increased virus replication and viruria (Leung et al. 2005).

Leung et al. proposed a model of HC in allogenic HSCT. Bladder-toxic agents usually damage the uroepithelium, which may result in an environment favoring the replication of BK virus. Then, the patient’s immunosuppression allows viral reactivation and replication. Alloimmune attack by donor lymphoid cells against BK viral antigens perpetuates the mucosal damage (Leung et al. 2005).

Schechter et al. reported that the sonographic findings of HC associated with BK virus following HSCT is characterized by multiple small mural nodules in a background of diffuse bladder wall thickening (Schechter et al. 2010).

To the best of our knowledge, there are only a few reports on the normal wall thickness of the urinary bladder. Kuzmic et al. reported that the respective normal detrusor thicknesses of the anterior, posterior, right lateral, and left lateral walls were 1.2 ± 0.4, 1.2 ± 0.4, 1.2 ± 0.4, and 1.2 ± 0.4 mm in healthy children younger than 18 years old, and they did not find a significant difference in the thicknesses of the four walls (Kuzmic et al. 2001). Based on their result, we measured the thickest point of the urinary bladder. If the site of measurement is fixed, then the BWT in nodular type HC might be measured in an area where it is thinner than average.

Many studies have suggested that multiple factors affect the development of HC. Consequently, we classified our patients according to the clinical settings and sonographic findings.

Classifying our patients by the type of bladder wall thickening, the mean disease duration and occurrence of hydronephrosis did not differ significantly between these two groups, in contrast to the use of bladder-toxic agents and BK viruria. The presence of BK viruria or the use of a bladder-toxic agent was not correlated with BWT, type of bladder wall thickening, or disease duration in our study. The occurrence of hydronephrosis was not correlated with BWT.

Nevertheless, we obtained several significant results. BWT on the initial sonography was positively correlated with the disease duration; the duration of disease increased with the BWT on initial sonography. Hydronephrosis occurred in 25.8 % of the patients with HC, and the incidence of hydronephrosis increased with the duration of HC. About 13.5 % of the patients who developed hydronephrosis underwent percutaneous nephrostomy for obstructive nephropathy and 25.0 % of the patients who developed hydronephrosis developed pyelonephritis. Eleven patients took cidofovir and 10 (90.9 %) of them did not develop hydronephrosis. We suggest that the use of cidofovir may help to prevent hydronephrosis and shorten the duration of HC in patients. The mean duration of HC without the occurrence of hydronephrosis was 36.5 days. A prospective randomized study of this is warranted.

In this study, all of the patients given cyclophosphamide as conditioning chemotherapy were also given Mesna. Eighteen patients used Mesna as prophylaxis for HC. Mesna binds acrolein, the urotoxic metabolite of cyclophosphamide and ifosfamide, in the urinary system and blocks acrolein from entering uroepithelial cells. Experimental and clinical studies have clearly demonstrated that Mesna is effective against cyclophosphamide-induced HC (Haselberger and Schwinghammer 1995). Nevertheless, about 5 % of the patients treated with cyclophosphamide or ifosfamide develop HC (Etlik et al. 1997; Shepherd et al. 1991). None of the patients given the bladder-toxic agent busulfan were given Mesna. No prophylactic drug for busulfan is known because the roles of busulfan and its metabolites are not clear. Hence, any patient given a bladder-toxic agent needs to be watched carefully, and if these patients complain of urinary symptoms, further evaluation, such as sonography, should be performed.

There were several limitations to our study. We calculated the volume of the bladder if it appeared to have collapsed. When the volume of the bladder was less than 50 % of its normal estimated capacity, the sonography examination was delayed for a few hours. Since this study was retrospective, the bladder volume was not measured in every case. An accurate estimation of volume was not always possible, although we rechecked the calculation, because our patients were children with frequency and dysuria. Therefore, this study had a limitation in terms of the standardization of bladder filling status.

## Conclusion

In conclusion, in patients with HC after HSCT/BMT, the BWT on the initial sonography may correlate with the symptom duration, and the duration of disease correlates with the occurrence of hydronephrosis.

## Methods

### Patients

Our Institutional Review Board approved this retrospective study and waived the requirement for informed consent. The clinical course and sonography of HC patients who underwent HSCT/BMT at our hospital between May 2009 and July 2014 were reviewed. All children were younger than 17 years of age and the medical charts and sonographic images of 31 patients were reviewed retrospectively.

The following data were collected for analysis: patient age, sex, and underlying disease, the day of HSCT/BMT, use of a bladder-toxic agent in a conditioning regimen, dates of HC diagnosis and normalized urinalysis, symptom duration (number of days), and urine PCR results for BK virus. Quantitative PCR for BK virus was performed on urine samples collected before HSCT/BMT and when HC developed after transplantation.

We also reviewed the initial and serial sonographic findings, including BWT, the type of BWT, and presence of hydronephrosis or pyelonephritis. Follow-up sonographic exams were performed at 1-week intervals until complete resolution of any microscopic hematuria and urinary symptoms.

We correlated the sonographic appearance with the clinical manifestations of HC for each patient.

### Definitions

#### HC, onset of HC, duration of disease

Droller et al. proposed a grading system for HC in which grade I HC is defined as the presence of microscopic hematuria and urinary symptoms such as frequency, urgency, or dysuria (Droller et al. 1982). Therefore, we selected patients with HC if the patient had at least microscopic hematuria (grade I HC) and urinary symptoms. Eleven patients had gross hematuria. We defined the onset of HC as the number of days from the date when the patient underwent HSCT/BMT to the date on which HC was diagnosed. We defined the duration of disease as the number of days from the date of diagnosis of HC to the date on which the patient’s urinalysis normalized. In other words, symptom duration was defined as the number of days with microscopic hematuria on urinalysis.

Microscopic hematuria was defined as >3 red blood cells (RBCs) per high-power field on two of three specimens, and normalized microscopic hematuria was defined as <3 RBCs per high-power field on urinalysis.

#### BWT and type of BWT on sonography

Abnormal bladder wall thickening was defined as wall thickness >3 mm. The BWT was measured by the radiologists who performed the sonographic examination. In our clinic, the BWT is routinely reported if patients have urinary symptoms or an abnormal urinalysis. If these patients have follow-up sonographic examinations, the changes in BMT are usually reported.

In our clinic, kidney and bladder sonography are performed with bladder filling at least 3 h before the examination. If a patient does not meet this condition, the examination is delayed for a few hours. Even if the patient meets this condition, the examination might be repeated if the radiologist decides that the bladder is not fully distended. If the radiologist thought that the bladder had collapsed, the volume of the bladder was measured and the examination delayed. The urinary bladder was considered not fully distended when the bladder volume was less than 50 % of the normal volume for age. The volume of the bladder (*V*) in milliliters was calculated using the formula (Norgaard et al. 1998):

$$V = a \; \times \;b \; \times \; c\; \times \; 0. 5 5$$

where *a* is the bladder width, *b* is the anteroposterior diameter, and *c* is the craniocaudal diameter, all measured by sonography. The calculated volume of the bladder was compared with the normal estimated bladder capacity (EBC) for age, using the formula (Norgaard et al. 1998):

$${\text{EBC}} = \left( {{\text{age in years}} \; \times \; 30} \right) \, +\, 30$$

If the bladder volume was less than 50 % of the EBC, the measurement of BWT was delayed for a few hours.

On sonography, the BWT was measured as the hypoechoic layer sandwiched between two hyperechoic layers (the uroepithelium and perivesical tissue) at the thickest of four points in the urinary bladder, i.e., the anterior, posterior, right lateral, and left lateral walls.

**Fig. 2:**
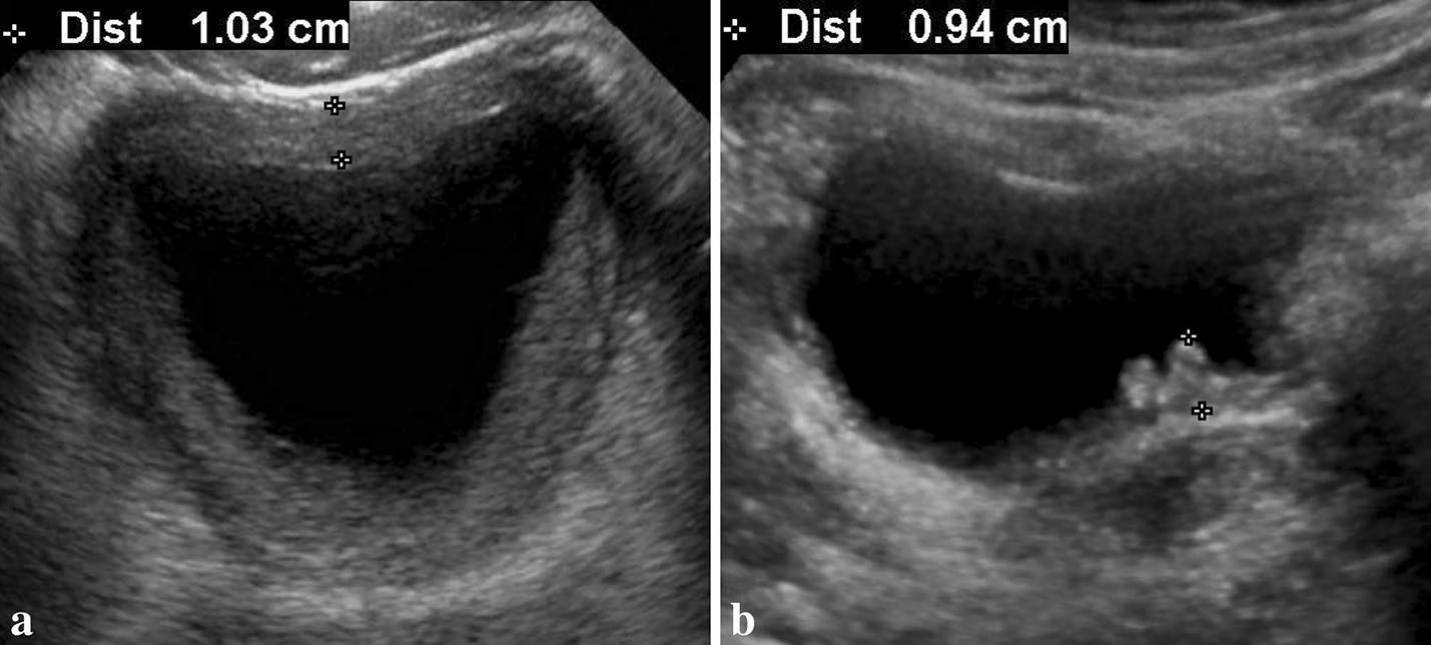
Two types of bladder wall thickening on bladder sonography of patients with hemorrhagic cystitis. **a** Diffuse type wall thickening; continuous, circumferential and even wall thickening of bladder. **b** Nodular type wall thickening; more than 3 mm-height of nodular protruding focus in a background of either continuous or discontinuous wall thickening of bladder

The BWT was classified into ‘diffuse’ and ‘nodular’ types. Diffuse bladder wall thickening comprised continuous, even wall thickening of bladder. In the nodular type, there were nodular protrusions >3 mm high in a background of either continuous or discontinuous bladder wall thickening (Fig. 2).

### Statistical analysis

Statistical analysis was performed using the SPSS for Windows software package (ver. 19.0; IBM Corp., Armonk, NY, USA).

A *p* value <0.05 indicated statistical significance. The Pearson correlation coefficient was calculated to determine the relationship between the BWT on initial sonography and the duration of disease. The Mann–Whitney *U*-test was used to compare the other variables.

## References

[CR1] Decker DB, Karam JA, Wilcox DT (2009). Pediatric hemorrhagic cystitis. J Pediatr Urol.

[CR2] Droller MJ, Saral R, Santos G (1982). Prevention of cyclophosphamide-induced hemorrhagic cystitis. Urology.

[CR3] Erard V, Storer B, Corey L, Nollkamper J, Huang ML, Limaye A, Boeckh M (2004). BK virus infection in hematopoietic stem cell transplant recipients: frequency, risk factors, and association with postengraftment hemorrhagic cystitis. Clin Infect Dis Off Publ Infect Dis Soc Am.

[CR4] Etlik O, Tomur A, Deveci S, Piskin I, Pekcan M (1997). Comparison of the uroprotective efficacy of mesna and HBO treatments in cyclophosphamide-induced hemorrhagic cystitis. J Urol.

[CR5] Gorczynska E, Turkiewicz D, Rybka K, Toporski J, Kalwak K, Dyla A, Szczyra Z, Chybicka A (2005). Incidence, clinical outcome, and management of virus-induced hemorrhagic cystitis in children and adolescents after allogeneic hematopoietic cell transplantation. Biol Blood Marrow Transpl J Am Soc Blood Marrow Transpl.

[CR6] Hale GA, Rochester RJ, Heslop HE, Krance RA, Gingrich JR, Benaim E, Horwitz EM, Cunningham JM, Tong X, Srivastava DK, Leung WH, Woodard P, Bowman LC, Handgretinger R (2003). Hemorrhagic cystitis after allogeneic bone marrow transplantation in children: clinical characteristics and outcome. Biol Blood Marrow Transpl J Am Soc Blood Marrow Transpl.

[CR7] Haselberger MB, Schwinghammer TL (1995). Efficacy of mesna for prevention of hemorrhagic cystitis after high-dose cyclophosphamide therapy. Ann Pharmacotherap.

[CR8] Hassan Z, Remberger M, Svenberg P, Elbander M, Omazic B, Mattsson J, Conrad R, Svahn BM, Ahlgren A, Sairafi D, Aschan J, Le Blanc K, Barkholt L, Ringden O (2007). Hemorrhagic cystitis: a retrospective single-center survey. Clin Transplant.

[CR9] Islam R, Isaacson BJ, Zickerman PM, Ratanawong C, Tipping SJ (2002). Hemorrhagic cystitis as an unexpected adverse reaction to temozolomide: case report. Am J Clin Oncol.

[CR10] Kimura M, Tomita Y, Morishita H, Takahashi K (1998). Presence of mucosal change in the urinary bladder in nonhematuric patients with long-term exposure and/or accumulating high-dose cyclophosphamide. Possible significance of follow-up cystoscopy on preventing development of cyclophosphamide-induced hemorrhagic cystitis. Urol Int.

[CR11] Kuzmic AC, Brkljacic B, Ivankovic D (2001). Sonographic measurement of detrusor muscle thickness in healthy children. Pediatric Nephrol (Berlin, Germany).

[CR12] Leung AY, Yuen KY, Kwong YL (2005). Polyoma BK virus and haemorrhagic cystitis in haematopoietic stem cell transplantation: a changing paradigm. Bone Marrow Transplant.

[CR13] Manikandan R, Kumar S, Dorairajan LN (2010). Hemorrhagic cystitis: a challenge to the urologist. Indian J Urol IJU J Urol Soc India.

[CR14] McCarville MB, Hoffer FA, Gingrich JR, Jenkins JJ (2000). Imaging findings of hemorrhagic cystitis in pediatric oncology patients. Pediatr Radiol.

[CR15] Norgaard JP, van Gool JD, Hjalmas K, Djurhuus JC, Hellstrom AL (1998). Standardization and definitions in lower urinary tract dysfunction in children. International Children’s Continence Society. Br J Urol.

[CR16] Ribeiro RA, Freitas HC, Campos MC, Santos CC, Figueiredo FC, Brito GA, Cunha FQ (2002). Tumor necrosis factor-alpha and interleukin-1beta mediate the production of nitric oxide involved in the pathogenesis of ifosfamide induced hemorrhagic cystitis in mice. J Urol.

[CR17] Ringden O, Ruutu T, Remberger M, Nikoskelainen J, Volin L, Vindelov L, Parkkali T, Lenhoff S, Sallerfors B, Ljungman P (1994). A randomized trial comparing busulfan with total body irradiation as conditioning in allogeneic marrow transplant recipients with leukemia: a report from the Nordic Bone Marrow Transplantation Group. Blood.

[CR18] Schechter T, Liebman M, Gassas A, Ngan BY, Navarro OM (2010). BK virus-associated hemorrhagic cystitis presenting as mural nodules in the urinary bladder after hematopoietic stem cell transplantation. Pediatr Radiol.

[CR19] Schoenike SE, Dana WJ (1990). Ifosfamide and mesna. Clin Pharm.

[CR20] Shepherd JD, Pringle LE, Barnett MJ, Klingemann HG, Reece DE, Phillips GL (1991). Mesna versus hyperhydration for the prevention of cyclophosphamide-induced hemorrhagic cystitis in bone marrow transplantation. J Clin Oncol Off J Am Soc Clin Oncol.

[CR21] Stillwell TJ, Benson RC (1988). Cyclophosphamide-induced hemorrhagic cystitis. A review of 100 patients. Cancer.

[CR22] Suzuki T, Yasumoto M, Shibuya H, Suzuki S (1988). Sonography of cyclophosphamide hemorrhagic cystitis: a report of two cases. J Clin Ultrasound JCU.

[CR23] Talar-Williams C, Hijazi YM, Walther MM, Linehan WM, Hallahan CW, Lubensky I, Kerr GS, Hoffman GS, Fauci AS, Sneller MC (1996). Cyclophosphamide-induced cystitis and bladder cancer in patients with Wegener granulomatosis. Ann Intern Med.

[CR24] Vogeli TA, Peinemann F, Burdach S, Ackermann R (1999). Urological treatment and clinical course of BK polyomavirus-associated hemorrhagic cystitis in children after bone marrow transplantation. Eur Urol.

[CR25] Vose JM, Reed EC, Pippert GC, Anderson JR, Bierman PJ, Kessinger A, Spinolo J, Armitage JO (1993). Mesna compared with continuous bladder irrigation as uroprotection during high-dose chemotherapy and transplantation: a randomized trial. J Clin Oncol Off J Am Soc Clin Oncol.

